# The current state of artificial intelligence-augmented digitized neurocognitive screening test

**DOI:** 10.3389/fnhum.2023.1133632

**Published:** 2023-03-30

**Authors:** Chananchida Sirilertmekasakul, Wanakorn Rattanawong, Assawin Gongvatana, Anan Srikiatkhachorn

**Affiliations:** Faculty of Medicine, King Mongkut’s Institute of Technology Ladkrabang, Bangkok, Thailand

**Keywords:** dementia, cognitive assessment, computerized screening test, artificial intelligence, machine learning, computer vision, automated speech recognition

## Abstract

The cognitive screening test is a brief cognitive examination that could be easily performed in a clinical setting. However, one of the main drawbacks of this test was that only a paper-based version was available, which restricts the test to be manually administered and graded by medical personnel at the health centers. The main solution to these problems was to develop a potential remote assessment for screening individuals with cognitive impairment. Currently, multiple studies have been adopting artificial intelligence (AI) technology into these tests, evolving the conventional paper-based neurocognitive test into a digitized AI-assisted neurocognitive test. These studies provided credible evidence of the potential of AI-augmented cognitive screening tests to be better and provided the framework for future studies to further improve the implementation of AI technology in the cognitive screening test. The objective of this review article is to discuss different types of AI used in digitized cognitive screening tests and their advantages and disadvantages.

## Introduction

According to the World Health Organization (WHO), approximately 50 million people are suffering from dementia and the number of cases is rising by approximately 10 million every year (World Health Organization, 2022). It has been predicted that the number of people with dementia will be tripled by 2050 (Alzheimer’s Disease International, 2021). The gradual decline of cognitive function leads to a wide range of displeasing behavioral and psychological problems, which eventually could lead to both financial and emotional burdens to the patients, caregivers, and family members (Chiao et al., 2015). Since AD is a neurodegenerative disease, there is no definite treatment available. Current pharmacological interventions are mostly symptomatic treatment, meaning that the medications are only beneficial when the disease has already been aggravated to a certain level (Yiannopoulou and Papageorgiou, 2013). Therefore, early diagnosis is of utmost importance for a patient’s access to care and support.

In recent years, the popularity of data-driven technology like artificial intelligence (AI) has massively increased in multiple fields, especially in cognitive neuroscience (Yu et al., 2018). Numerous pieces of evidence have shown the ability of AI to improve patient care, specifically aiding disease diagnosis, and supporting rational clinical decision-making. At present, several studies have also adopted this technology in developing neurocognitive screening tests, aiming to further advance the digital version of these tests by improving the accuracy of the scoring system and reducing the majority of the disadvantages of the paper-based version. Multiple implementations of the AI methods have been used in different test batteries. This review article will be focusing on the application of three AI implementations: machine learning (ML) and deep learning (DL), computer vision, and automatic speech recognition (ASR). This paper will also discuss the advantages and limitations of the digitized cognitive screening test.

## Methodology

The information about AI-augmented cognitive screening tests was retrieved from PubMed (MEDLINE) database by searching multiple keywords: “dementia or cognitive impairment,” “computerized or digitized cognitive screening test,” “artificial intelligence or AI,” “machine learning,” “deep learning,” “computer vision,” and “automated speech recognition.”

Moreover, the Boolean AND and OR operators were used for the following search strings: dementia AND computerized cognitive screening test AND (artificial intelligence OR machine learning OR deep learning OR computer vision OR automated speech recognition). The literature search was carried out from January 2000 to December 2022. The papers selected were based on the relevance of the application of AI technology to a cognitive screening test.

## AI-augmented neurocognitive screening tests

### The conventional neurocognitive tests

Among most of the neurocognitive screening tests, the similarities among them are that they are paper-based and are bound to the physical clinical settings, restricting the protocol to be administered by specialized medical personnel only (Thabtah et al., 2019). The medical personnel are required to explain the protocol, supervised test execution, and assigned the score after test completion (Lunardini et al., 2020), showing that the test relied hugely on the administrators who could raise the concern of biases and inter-rater reliability. Restricting the test administration in clinical settings is, too, another issue. Older adults might not be motivated to undergo a cognitive assessment with healthcare personnel or have difficulties in accessing healthcare services (Chan et al., 2018), contributing to the main problem of delayed detection of cognitive impairment (Lunardini et al., 2020). Especially during the pandemic, the implementation of social restrictions and self-isolation as preventive measures for COVID-19 had undeniably created obstacles in healthcare management, including the administration of cognitive screening tests (Toniolo et al., 2021).

### Adaptation of cognitive assessment during the COVID-19 pandemic

The coronavirus pandemic has markedly raised the demand for remote cognitive tests to solve the problem. Telemedicine, remote medical care using the means of telecommunications technology, appeared to be the practical solution for this problem (Sene et al., 2015). Because of its special characteristic to treat patients without their physical presence at a hospital *via* videoconferencing, telemedicine has been used to overcome distance barriers and to improve the accessibility to the healthcare service. Multiple studies were carried out to find the agreement and correlation between the original paper-based and the digitized versions. Carotenuto et al. (2021) has performed a systematic review to clarify whether the traditional face-to-face (FTF) neuropsychological tests, mainly Mini-Mental State Examination (MMSE), are reliable *via* telemedicine. A total of 68 papers were included. The result has shown that MMSE scores were not different when tests are administered either *via* FTF or videoconference modalities, and only negligible minor changes in the scoring system were required. Other neuropsychological tests such as the Token Test, and the Comprehension of Words and Phrases (ACWP), also showed high reliability between the two modalities.

Even though these telemedicine versions can reduce the transportation barrier, the test administration remains strictly to the medical personnel. To remove all these limitations from the paper-based version, digitized versions of cognitive screening tests have been developed, by having the main goal of reducing the reliance on medical personnel and increasing the accessibility of the test.

The table below summarized the AI features used, and the advantages and disadvantages of all the studies mentioned in this review article (Table 1).

**TABLE 1:** Summarization of studies using AI-augmented digitized neurocognitive screening test.

No.	References	Type of test	AI features used	Advantages	Disadvantages
1	Chen et al., 2020	Clock drawing test (CDT)	DL	Information was stored in secured cloud storage Helped reassuring physicians’ opinions Keep track of patient’s mental status *, **	Unable to interpret complicated drawings correctly ***
2	Park et al., 2020	Pentagon drawing	DL	Increased the accuracy of cognitive function assessment in PD Able to measure multiple tremor parameters Age-friendly interface design Information was stored in secured cloud storage Can be used as personal lifelog *,**	***
3	Binaco et al., 2020	CDT	ML	Higher test-retest reliability and sensitivity than the gold standard method *	Multi-classification of >2 groups were not as accurate as binary classification More costly Rely on specific classification algorithms
4	Sato et al., 2022	CDT	DL	Sufficient reliable training data set from NHATS high prediction accuracy, especially in participants with executive dysfunction	Grading criteria from NHATS was not provided Participants demographic background were unknown Increased easiness to cheating
5	Kaiser et al., 2014; Maltby et al., 2020	Salzburg dementia test prediction (SDTP)	DL	Administration time less than 5 min High sensitivity with the new cutoff value Equivalent convergent validity to ACE-III	The model was trained to classify the patients into binary groups only: demented and non-demented Cannot discriminate between normal control subjects and those with mild cognitive impairment
6	Rutkowski et al., 2020	Behavioral reaction time, and emotional arousal/valence responses detection	ML	High accuracy New methods for early determination of cognitive decline Low cost	The model was trained to classify the stages of MCI into binary groups only
7	Hafiz et al., 2019	Screen for cognitive impairment in psychiatry (SCIP) test	ASR	Easy to use Showed correlation with the original test Acceptable concurrent validity Relatively low word error rate – allowing real-time assessment of verbal memory	Limited only to the trained language(s) Could not grade spelling accuracy
8	König et al., 2018	Semantic verbal fluency task	ASR	Very strong correlation with the original version Relatively low word error rate Provide possibility of fully automated pipeline Can collect more useful additional measurements Keep track of their patient’s mental status *, **	Limited only to the trained language(s)
9	Toth et al., 2018	Acoustic parameters (hesitation ratio, speech tempo, length of silent and filled pauses, length of utterance)	ASR, ML, and DL	Extract non-verbal acoustic features Novel methods to detect early cognitive decline *	Limited only to the trained language(s) Large word error rates for certain languages
10	Kantithammakorn et al., 2022	Language fluency test battery from Montreal cognitive assessment (MoCA)	ASR	Novel ASR utilization for assisting a phonemic fluency task in Thai Provided new techniques that can be used as a baseline for future ASR integration in other speech recognition domains Provided a framework for future ASR-assisted Thai cognitive screening tests Low word error rate (WER)	Limited only to the trained language(s) Limited tone detection due to insufficient pitch difference data
11	Schultebraucks et al., 2020	Facial, movement, and speech analysis	CV and ML	Novel and easier methods to detect early cognitive decline Features from free speech interviews can be utilized to assess multiple domains of cognitive functioning Higher accuracy and test-retest reliability than original cognitive assessments Does not require structured clinical interview Helped eliminating practice effects *, **	Could not define the definite relationships among the selected features Could not truly understand how the algorithm works Could not interpret the result by using single extracted features Required multiple variables for interpreting the results
12	Jiang et al., 2022	Facial emotions analysis	CV and DL	Independent of race, sex, education level, eye movement, and the existence of depression Extract non-verbal features Novel and easier methods to detect early cognitive decline Novel and easier methods to detect early cognitive decline Features from free speech interviews can be utilized to assess multiple domains of cognitive functioning Higher accuracy and test-retest reliability than original cognitive assessments Does not require structured clinical interview Helped eliminating practice effects *, **	Facial emotions might be affected by individuals’ stress Required baseline reference emotion expression Could not differentiate underlying etiologies of cognitive impairment
13	Jiang et al., 2022	Facial emotions analysis	CV and DL	Independent of race, sex, education level, eye movement, and the existence of depression Extract non-verbal features Novel and easier methods to detect early cognitive decline *	Facial emotions might be affected by individuals’ stress Required baseline reference emotion expression Could not differentiate underlying etiologies of cognitive impairment

*Automated, convenient, low cost, and reduced raters’ bias.

**Remote control, increase accessibility, available on any mobile device, and reduces medical personnel’s workload.

***Required large datasets, required professionals to gather, and label the training dataset. ML, machine learning; DL, deep learning; ASR, automatic speech recognition; CV, computer vision; CDT, clock drawing test; ACE-III, Addenbrooke’s Cognitive Examinations.

### Machine learning and deep learning

AI technology consists of many fields, and one of the most famous fields is ML. ML is the computer algorithm that allows the machine to “learn” and detect patterns from the input data, rather than “being instructed,” giving the output as an improvement of machine automaticity (Graham et al., 2020; Zhai et al., 2020). DL is a subclass of ML that aims to imitate human brain function, by training the model to think systematically as humans do. This goal was accomplished by simulating multilayer artificial neural networks (ANNs) to mimic real human neural connections. ANNs can independently figure out and extract specific features from the provided raw input data, allowing more detailed information to be obtained (Delua, 2021). This allows the decision-making process to be more precise and more accurate, compared to the decision made by humans (Lee et al., 2017).

### Neurocognitive tests using machine learning and deep learning

The AI models had been applied to different test batteries to increase the efficacy and accuracy of the tests. Binaco et al. (2020) and Chen et al. (2020) performed studies on the AI-assisted Digital Clock Drawing Test (CDT), which is the test that is used to detect the presence of visuospatial, attention, or executive dysfunctions of the individuals, to differentiate patients into different groups. The results showed its impressive ability to classify non-MCI, MCI, and AD patients with an accuracy above 90%. More recently, Sato et al. (2022) also developed AI-assisted CDT by training the deep neural network (DNN) model with a large number of training data of over 40,000 drawings obtained from a large cohort of older adults from the National Health and Aging Trends Study (NHATS). The results have shown an accuracy of approximately 90% for identifying participants with a declined executive function and up to 77% accuracy for identifying participants with probable dementia. This result not only suggested its potential to be the “mass screening test” for ruling in those with executive dysfunction or with probable dementia but also verified the reproducibility of the previous DNN-based CDT scoring models.

Other than CDT, the pentagon drawing test (PDT) and Trail Making Test (TMT) had also been digitized. Park et al. (2020) upgraded the conventional PDT to be more sensitive to assess cognitive function in Parkinson’s disease (PD) pants by utilizing both AI and sensor technologies. The DL algorithm, U-Net, was used to detect and classify the drawings, and a mobile sensor was used to collect additional data on hand tremors. And instead of classifying PDT into ordinary accurate and inaccurate groups, they alternatively used AI to regulate the scoring system for the test by detecting the number of angles (0–4 points), the intersection between the two pentagons (0–4 points), closure/opening of the figure (0–2 points), and tremors detected (0–1 points), which give a total score of 11. This caused their test to be even more sensitive to detect cognitive impairments in PD, compared to the paper-based PDT.

Moreover, DL can also be applied as a score predictor. Kaiser et al. (2014) had developed the new three-question dementia screening test, the Salzburg dementia test prediction (SDTP), aiming to reduce the total administration time to less than 5 min and lowering the rate of false positives compared to other short-form of cognitive screening tests. Test batteries include asking the exact weekday and year, and spelling the given word backward (RADIO). ANNs were then used to predict the MMSE score from the three answers received. The result has shown an impressive sensitivity of 94% with the new cutoff value at 25/30, and a specificity of 68%. Maltby et al. (2020) also carried out a validation study to verify whether SDTP can be used as a reliable cognitive screening tool for dementia patients. SDTP was compared to the Addenbrooke’s Cognitive Examinations (ACE-III) and the result has shown that SDTP and ACE-III have an equivalent convergent validity to MMSE, meaning that this MMSE-derived AI-augmented brief cognitive screening test is impressively comparable to the standard qualified cognitive screening test.

### Neurocognitive test using computer vision

Computer vision is another field of AI that is developed aiming to mimic the real human visual system (IBM, 2021). It mainly utilizes the DL algorithm “convolutional neural network (CNN)” which enables computers to acquire meaningful information from the provided digital images or videos, learn independently, and interpret them in a meaningful manner (IBM, 2021). The algorithm consists of multiple neural network layers, in which different layers will acquire different types of data. The initial layers will be responsible for identifying and learning basic data, such as straight lines and curves, the next layers then acquire more complex data such as shapes and colors of the images. The higher-level layers of the algorithm will then aggregate all the information, allowing the algorithm to understand the whole picture (Esteva et al., 2019). As the system was trained to detect and analyze numerous media at a time, its performance can easily surpass human ability and provide huge advantages to many fields of work. In the neuropsychiatric field, digital phenotyping, such as patients’ behaviors, facial expressions, or emotional responses could be taken into account for clinical judgment (Leo et al., 2020).

An example of a computer vision application was from Schultebraucks et al. (2020) they utilized the computer vision AI model and voice analysis to predict cognitive function in trauma survivors. The main objective of this study was to predict patients’ cognitive functions through passive data sources, like facial expressions, movement, and speech characteristics of the patients. The results had shown that digital biomarkers were suitable for predicting patients’ cognitive functions as high diagnostic accuracy was provided. In another study from Jiang et al. (2022) utilized a computer vision-based deep learning model for analyzing facial emotions in subjects with cognitive impairment. They separated participants into two groups according to their MoCA scores: cognitively impaired (CI) and cognitively unimpaired (CU). They were asked to perform a Visuospatial Memory Eye Tracking Test, a passive viewing test that asked participants to view the images displayed on the screen, and their reactions were recorded. The results from computer vision analysis had shown that CI participants expressed significantly fewer positive emotions, more negative emotions, and higher facial expressiveness during the test. The advantage of this facial emotion analysis was that it allowed effective differentiation of CI from CU participants, with a large independent from sex, race, age, education level, mood, and eye movements. The findings provide quantitative and comprehensive evidence that the expression of facial emotions is significantly different in people with cognitive impairment and suggests this may be a useful tool for passive screening of cognitive impairment.

### Automatic speech recognition technology

Another well-known AI subfield is natural language processing (NLP). It is a linguistic subfield that allows computers to read and interpret human languages. One of the most commonly used NLP subtypes is ASR, also known as speech-to-text (STT). STT is the technology that allows the computer to recognize and understand human speech through direct speaking to the computer interface (Tröger et al., 2019; Jiang et al., 2022). The main advantages of ASR are that it can solve the problems of user-interface unfriendliness and technology unfamiliarity. This is because instead of requiring the patients to type down the answers, ASR could be used to provide a more comparable mode of answering to the test administrator. The patient can speak directly to the mobile phone and AI will synthesize them into words. This would capably reduce the bias and technology-induced anxiety arising from patients’ unfamiliarity with mobile devices, especially among the elderly.

Multiple studies had utilized ASR technology in the digitized cognitive screening test, allowing individuals to answer directly to the computer interfaces. In a study by Tröger et al. (2019) applied ASR to their telephone-based dementia screening test, aiming to assess semantic verbal fluency (SVF) in dementia patients. Similarly, König et al. (2018) also applied ASR to the SVF test for qualitative screening for neurocognitive impairment in both AD and MCI patients. Toth et al. (2018) developed a neuropsychological screening test to detect cognitive impairments by analyzing speech production during performing a memory task. ASR was utilized for extracting acoustic parameters, including hesitation ratio, speech tempo, and length of utterance. Their results showed significant differences between healthy individuals and MCI patients in terms of their acoustic features of delayed recall. Another example is a study by Hafiz et al. (2019) who developed their internet-based cognitive assessment tool (ICAT) from the screen for cognitive impairment in psychiatry (SCIP) test and Google’s ASR was utilized for the speech-based answers. This study has evaluated the accuracy rate of Google’s ASR and the result has shown insignificant error rates in both Danish and English languages, providing promising backup evidence for the future development of ASR-assisted neurocognitive tests. All these studies had shown that the implementation of ASR technology enhances the feasibility of the preferred self-administrable characteristics of the cognitive screening test and allows a wider age range of the population to perform the test.

Recently Kantithammakorn et al. (2022) developed a novel ASR-assisted Thai language fluency test battery in the Montreal cognitive assessment (MoCA). As the Thai language is a tonal language, the challenge for the developmental process was to detect and differentiate words with similar tones. Multiple ASR techniques were required to train this acoustic model to be resilient to background noise and to be responsive to the variation of tone and voice quality. One of the main limitations of this study is the limited training dataset. The Thai speech data of MCI patients were unavailable as it was the first attempt to collect data from the MoCA assessment in digital format for the Thai language. Nevertheless, this study has provided a framework for future ASR-assisted Thai cognitive screening tests and provided new techniques that can be used as a baseline for future ASR integration in other speech recognition domains.

## Advantages and limitations of computerized cognitive assessment

As the majority of the cognitive screening tests are considered to be a repetitive task as they have a well-constructed test administration workflow pattern and standard scoring criteria to follow, the implementation of the automated scoring system (ASS) is considered to be very useful and provides multiple advantages. First, the ASS is considered to be resource-efficient as the clinicians are not required to administer the test by themselves, allowing them to reduce their workload and concentrate more on other AI-irreplaceable tasks. Second, ASS could effectively improve the scoring consistency and reduce the risk of interrater incongruence as every test will be graded according to a single standardized scoring algorithm (Chan et al., 2018; Monsch et al., 2019). Additionally, the availability of the digitized version on the digital platform can increase accessibility to the population, especially those living in healthcare-unreachable areas, allowing the cognitive screening test to enhance its intention of early identifying individuals with cognitive impairments and also to reduce the inequality of health services field (World Health Organization, 2020). Similarly, electronic tests can provide additional useful data that could not be retrieved from the paper-based version, allowing a wider variety of questions to be created to assess more diverse cognitive domains and other sensory and motor functions (Kokubo et al., 2018; Lauraitis et al., 2020; Lunardini et al., 2020). For the advantages of ASR-assisted neurocognitive, it would provide a more comparable version to the original version of having a speech-based test administration. And comparing to typing-based answers, speech-based answers are better at controlling the practice effect as it has been shown that typing could significantly influence human short-term memory (Hafiz et al., 2019). Therefore, ASR could potentially help prevent overrating test scores.

Despite all the advantages of the computerized version of cognitive assessments, there are still multiple limitations and disadvantages. Socioeconomic barriers are one of the main restrictions. The differences in cultural backgrounds, educational level, availability of the internet, and the afford to pay for the hidden costs of technology usage, such as internet access and insurance policies, among individuals can lead to significant unequal access to healthcare and prevent the utilization of the digital technology for the healthcare system (Chinner et al., 2018; Hafiz et al., 2019; Lunardini et al., 2020). Sarkar et al. (2011) has studied the use of the internet-based patient portal on their participation in medical management among the elderly with chronic disease, diabetes mellitus. Even with adequate internet and computer access, they have found that individuals with ethnic minorities, older ages, and lower educational attainment were less engaged with online patient portals to participate in the chronic disease management (Sarkar et al., 2011). From this study, we can conclude that despite having adequate access to the internet and technology, low digital and health literacies still caused an ineffective use of health applications (Eruchalu et al., 2021).

Another potential contribution to technology acceptance was technology literacy and familiarity. Due to the gap in a generation, elderly patients are more likely to be unfamiliar with technology usage, and some even have anxiety or negative attitudes toward them (Lauraitis et al., 2020). Czaja et al. (2006) also stated that computer anxiety is often found in the elderly which can consequently increase the stress level and reduce attention which can result in a false judgment or misinterpretations of the test. Hays et al. (2019) and Graham et al. (2020) also found that smartphone familiarity is one of the sources of bias in assessing cognition because their study suggested that individuals with higher familiarity with iPads and other tablets may perform better in certain areas of cognitive assessment than those who are not. These challenges commanded developers and physicians to find ways to enhance technology adoption by these users. The difficult part of technology is likely derived from these individuals’ ability to grasp the technical language and familiarity with user interfaces on smartphones. Monsch et al. (2019) also pointed out the problem of inaccurate perceptions of how elderly or demented patients used mobile technology can lead to ineffective designs, which can result in an unsuccessful creation of a digitized tool for cognitive assessment.

Not every digitized screening test was compared with the standard paper-based version and validated in a large population, causing a decrease in the generalizability, reliability, and validity of the test (Lunardini et al., 2020). The assumption of similar validity between the two versions should not be simply implied as Lunardini et al. (2020) had performed a comparison test between the paper-and-pencil and digitized versions of the Bells test, and the result had shown that the two versions of Bells test only showed a weak correlation. There were many contributing factors leading to different outcomes, including some modified questions, familiarity with the hardware (Monsch et al., 2019), or even minor interface issues like a decrease in visual acuity due to smaller font size on the screen (Lauraitis et al., 2020). Therefore, there was an increase in the demand for studies on distinct normative data for the newly digitized version (Monsch et al., 2019) and validation studies on the large heterogeneous populations for assessing the effectiveness and reliability of these technology-based tools (Mandal et al., 2015).

The security of patients’ information was also one of the major concerns for the digitized test. As their records were transferred *via* network and stored in the online platform, they were more vulnerable to leakage and other potential threats (Mandal et al., 2015; Chinner et al., 2018). Therefore, the confidentiality and privacy of a patient’s data must be taken into serious consideration.

Lastly, many applications could not create full self-administered tests because some items required supervisors to ensure the correct understanding of the test commands (Lauraitis et al., 2020). This is because an uncontrolled environment and limitation in cognitive or sensory functions can lead to improper execution, and ultimately resulting in the wrong result of the test (Lunardini et al., 2020).

## Author’s opinion

Due to the rapid growth of the elderly society and in conjunction with the COVID-19 pandemic situation, the demand for telehealth has significantly increased. Expansive impacts and the number of patients suffering from neurodegenerative diseases were significant indicators notifying us of the importance of developing these traditional paper-based screening tests into a more-convenient version to increase the availability and modulate the need for social distancing. The future direction of digitized cognitive screening tests should be aiming to have these four properties: elderly-friendly, inclusive, independently automated, and secured.

The “elderly-friendly” interface for the elderly should be detailed about the components relating to sensorial components, including visual and auditory elements, as most of them would be having a declined sensation. A “minimalist design,” also known as the utmost simplicity, is also one of the criteria for composing a successful age-friendly interface. By having concise instruction, reducing complex actions required to interact with the devices, and making their journey as less rough as possible, the concept of “less is more” would be achievable.

Also, as currently we are stepping forward to the digital era of the healthcare system, multiple innovations such as telemedicine and AI-infused healthcare technology were rapidly developed and were already taken into implementation. Preparing the elderly readiness to receive the new means of healthcare delivery could help boost the effectiveness of medical management. A digital literacy training class for the elderly by teaching them how to use different devices or an introductory course to digital healthcare could be a realistic choice to increase the engagement of the elderly with technology and to reduce their negative attitudes toward technology. The institute of museum and library services (IMLS) had established the framework of action for building a digital community and they also suggested that the healthcare centers should collaborate with community colleges, public libraries, and other community-based organizations to develop digital literacy training and skills-building programs to address digital and health literacy gaps for the population, especially elderly (Becker et al., 2012).

It is a consensus that computerized cognitive screening tests can reduce transportation costs and barriers for patients, allowing the expansion of healthcare accessibility to reach out to those who live farther away from the healthcare center. However, as the main medium for a computerized cognitive screening test to operate is through the internet, the drawback of this fact is that there will still be an accessibility barrier to places that lack of access to the internet. The future direction for the development of digital cognitive screening tests or other health assessments should be aiming at improving accessibility, affordability, and equity, especially for individuals with lower socioeconomic and educational levels. For improving the accessibility and affordability of the internet, IMLS had recommended the following ways: (1) Provide access to electronic information about community resources and services at strategic locations such as community-based organizations offering social service assistance. (2) Collaboration with the Federal Communications Commission to set goals and milestones for every household to achieve access to high-speed Internet. (3) Providing programs or campaigns that subsidize monthly Internet subscription costs for low-income households (Becker et al., 2012).

Even though most digitized cognitive screening tests claimed to be automated, not every test could be administered independently without a supervisor. As many elderlies are living alone, the future versions of these tests should work on improving this property. At last, every available test should be mindful of patients’ confidentiality. Their information should be secured in reliable cloud storage. Not only about confidentiality, but these clouds would help retrieve back patients’ previous results, or they could transfer the information to other team members which would smoothen and improve the effectiveness of patient care.

## Conclusion

To summarize, up to the current date, multiple AI models have been adopted into cognitive screening tests, including both ML and DL detection and classification algorithms, NLP, and computer vision. These studies have shown multiple advantages, such as increasing the accessibility and availability of cognitive screening tests, reducing the requirement of the medical personnel to administer the test, and reducing the risk of interrater incongruence from traditional paper-based tests. Nevertheless, there were still multiple drawbacks and limitations to this computerized cognitive screening test, including low technology familiarity with the elderly, unproven reliability and validity, patient information security, and uncontrolled test administration protocol. Therefore, future research is required to reduce these limitations and improve the digitized cognitive screening test version.

## Author contributions

CS, WR, AG, and AS: conceptualization, writing—original draft, and review and editing. All authors contributed to the article and approved the submitted version.
